# 82786 Quantification of the Accuracy of Stereotactic Radiosurgery using Surface Guided Imaging with 3D Printed Head Phantoms

**DOI:** 10.1017/cts.2021.665

**Published:** 2021-03-30

**Authors:** Victoria Bry, Daniel Saenz, Evangelos Pappas, Niko Papanikolaou, Karl Rasmussen

**Affiliations:** 1 The University of Texas Health at San Antonio; 2 University of West Attica

## Abstract

ABSTRACT IMPACT: This work assesses clinical implementation of a surface guided imaging system to improve the accuracy radiation delivery for treatment of brain lesions using a patient CT derived head phantom. OBJECTIVES/GOALS: Advancements in radiotherapy design have made clinical demand for efficient and accurate methods to deliver stereotactic radiosurgery (SRS) for treatment of intracranial lesions. This study assesses the potential of using surface guided imaging for setup using a 3D patient specific head phantom. METHODS/STUDY POPULATION: A single isocenter, multiple metastases SRS plan was generated on a CT derived RTsafe Prime phantom made of tissue equivalent materials and a polymer gel insert. Five targets of varying diameters were treated with 8Gy of radiation using two different positioning techniques. The first gel insert was irradiated within the phantom according to internal alignment with standard orthogonal x-ray imaging while the second setup used surface guided imaging, based on external anatomy. 42 hours after irradiation, the phantom was scanned in a head coil using a 1.5T MRI. MR images were fused with the patient CT data and structure set to further evaluate calculated and measured dose distributions. RESULTS/ANTICIPATED RESULTS: Discrepancies in phantom setup according to standard orthogonal x-ray imaging compared to surface guided imaging demonstrated to be <1mm in each translational (vertical, longitudinal, and lateral) and angular (rot, roll, pitch) directions. The 3D gel inserts permitted spatial analysis to compare dose distributions of measured values to those calculated in a treatment planning system (TPS). 3D GI (Gamma Index) analysis showed good alignment in high dose regions and resulted in passing rates >94% (5%/2mm) and >87% (3%/2mm). Finally, 3 of 5 targets showed better 3D GI passing rates and less geometric offset for positioning with the surface guided imaging. DISCUSSION/SIGNIFICANCE OF FINDINGS: 3D spatial analysis of human like phantoms demonstrated that patient positioning according to external anatomy performed comparable to standard methods aligning to the internal anatomy, for a multiple met SRS treatment.

